# Preparation, characterization and antitumor activity evaluation of apigenin nanoparticles by the liquid antisolvent precipitation technique

**DOI:** 10.1080/10717544.2017.1399302

**Published:** 2017-11-08

**Authors:** Weiwei Wu, Yuangang Zu, Li Wang, Lingling Wang, Huimei Wang, Yuanyuan Li, Mingfang Wu, Xiuhua Zhao, Yujie Fu

**Affiliations:** a Key Laboratory of Saline-alkali Vegetation Ecology Restoration in Oil Field (SAVER), Ministry of Education, Alkali Soil Natural Environmental Science Center (ASNESC), Northeast Forestry University, Harbin, China;; b Key Laboratory of Forest Plant Ecology, Northeast Forestry University, Ministry of Education, Harbin, China

**Keywords:** Apigenin, apigenin nanoparticles, solubility, bioavailability, antitumor activity

## Abstract

The present work aimed to apply the liquid antisolvent precipitation (LAP) method for preparing the apigenin nanoparticles and thereby improving the solubility and bioavailability of apigenin. The different experimental parameters on particle size were optimized through central composite design (CCD) using the Design-Expert^®^ software. Under the optimum conditions, the particle size of the apigenin nanosuspension was about 159.2 nm. In order to get apigenin nanoparticles, the freeze-drying method was selected and the mannitol was used as a cryoprotectant. Then the solid state properties of the apigenin nanoparticles were investigated using scanning electron microscopy (SEM), differential scanning calorimetry (DSC), thermo gravimetric (TG), and X-ray diffraction (XRD). The results obtained displayed that the apigenin nanoparticles exhibited near-spherical shape and could be transformed into an amorphous form. In addition, the dissolving test, the bioavailability in rats, and the antitumor activity were also studied. The experimental results showed that the solubility of the apigenin nanoparticles were about 29.61 times and 64.81 times of raw apigenin in artificial gastric juice and in artificial intestinal juice, respectively, and the apigenin nanoparticles showed higher dissolution rates compared to raw apigenin, and was about 6.08 times and 6.14 times than that of raw apigenin in artificial gastric juice and in artificial intestinal juice. The oral bioavailability of apigenin nanoparticles was about 4.96 times higher than that of the raw apigenin, but the apigenin nanoparticles had no toxic effect on the organs of rats. In addition, the apigenin nanoparticles had a higher inhibition to HepG2 cells by lower IC50 than that of raw apigenin.

## 1 Introduction

**Figure 1.**
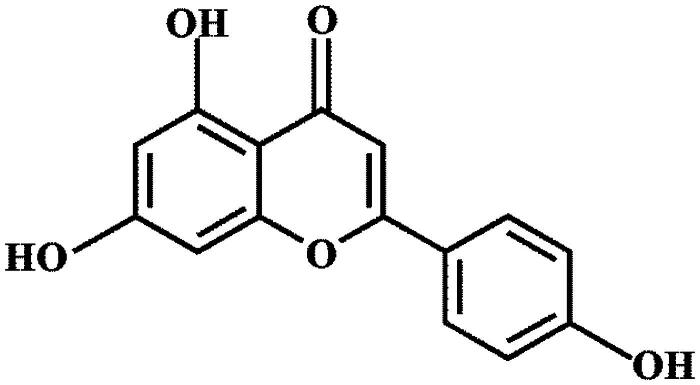
Chemical structure of apigenin.

Apigenin (5, 7-dihydroxy-2-(4-hydroxyphenyl)-4H-1-ben-zopyran-4-one, Figure 1), an active ingredients found in various vegetables and fruits, including parsley and onion (Li, 2014; Shen et al., 2014), has been reported to have numerous pharmacological effects. Apigenin can exert anti-inflammatory (Zhai et al., 2013; Zhang et al., 2013), antiviral (Qian et al., 2015; Zhao et al., 2016) and antioxidative effects (Kim et al., 2016; Telange et al., 2016), and can regulate differentiation of cells (Balasubramanian & Eckert, 2007) and so on. In addition, it also has an antiproliferative activity against liver, skin, pancreatic, neuroblastoma, breast, and colorectal cancer cell lines (Li et al., 2017) (Zhang et al., 2012; Ding et al., 2014). However, the apigenin is poorly absorbed with low bioavailability owing to poor water solubility by human body, which has severely limited its clinical applications and therapeutic efficiency.

In recent years, numerous studies have shown that micronization or size reduction is one of the effective formulation strategies to enhance the solubility and the dissolution rate of poorly water-soluble drugs (Merisko-Liversidge et al., 2003; Kesisoglou et al., 2007; Dong et al., 2010), and has attracted more and more attention. Based on Noyes–Whitney equation, the decrease of the particle size could increase the solubility and dissolution rate of poorly water-soluble drugs due to the increase of the effective particle surface area, and hence improve bioavailability (Kesisoglou et al., 2007). Nowadays, many studies set their aims at reducing particle size by means of nanonization and take advantage of the large specific surface areas afforded to improve the solubility and bioavailability of apigenin and some related references were summarized in Table 1. From the table, some effective micronization technologies have been used in reducing particle size of apigenin, including the bead milling techniques and the high-pressure homogenization (Al Shaal et al., 2011), the SmartCrystal combination technology (Al Shaal et al., 2010) (the combination method of the pearl milling and the high-pressure homogenization), and the supercritical fluid technique (Zhang et al., 2013) and so on. But these techniques need high-energy input and result in low particle size uniformity and metal contamination (Zhang et al., 2009). In addition, the supercritical-fluid technique also exists many problems including large equipment investment, wasted energy, and lower productivity (Zhe et al., 2007; Zhao et al., 2013), and so on. Moreover, the apigenin-loaded liposomes (Banerjee et al., 2015; Banerjee et al., 2017; Jin et al., 2017), the apigenin-loaded ethosome (Shen et al., 2014), the apigenin-loaded micelles (Zhai et al., 2013; Zhang et al., 2017) and the apigenin-loaded emulsions (Kim et al., 2016) of small particle size are also successfully prepared, but these nanoformulation strategies may have the problems of low entrapment efficiency and poor stability. The liquid antisolvent precipitation (LAP) technique is believed to be a promising technique in preparing nano/micro particles in recent years, compared with other micronization technologies, its process is relatively simple, easy to operate, lower cost, high yield, and has the potential to be applied in the pharmaceutical industry (Zu et al., 2013; Yadav & Kumar, 2014). Moreover, this technique has been successfully used to prepare several drugs, such as cilnidipine (Singh et al., 2014), rifampicin (Viçosa et al., 2012), trans-resveratrol (Kim et al., 2012), amphotericin B (Zu et al., 2013) and taxifolin (Zu et al., 2014) and so on. However, there have been no reports on the preparation of apigenin nanoparticles by the LAP technique so far. Therefore, the aim of this study is to apply the LAP technique to prepare apigenin nanoparticles for improving the solubility and dissolution of poorly water-soluble apigenin.

**Table 1.** Summary of reports available on production of Apigenin particles.

Type	Production techniques used	Particle sizeobtained (nm)	Water solubility	Oral bioavailability improved (AUC values)	Reference
Apigenin nanocrystals	Combination technology (CT), i.e. bead milling and subsequently high-pressure homogenization	413	–	–	Al Shaal et al., 2011
Apigenin nanocrystals	SmartCrystal combination technology	3–3000	–	–	Al Shaal et al., 2010
Apigenin nanocrystals	Supercritical antisolvent process	400–800	–	3.4 times	Zhang et al., 2013
Apigenin-loaded liposomes	Lipid film hydration method	103.20 ± 2.1	–	–	Banerjee et al., 2017
Apigenin-loaded liposomes	Lipid film hydration method (slight modification)	104.3 ± 1.8	–	–	Banerjee et al., 2015
Apigenin-loaded tocopherol derivative-containing D-alpha-tocopheryl polyethylene glycol 1000 succinate (TPGS) liposomes	Lipid film hydration method (slight modification)	118.6 ± 8.1	–	–	Jin et al., 2017
Apigenin-loaded ethosome	Conventional mechanical dispersion	90.99 ± 2.20	–	–	Shen et al., 2014
Apigenin-loaded polymeric micelles	Thin-film dispersion method	16.9	320.8 μg/mL	–	Zhai et al., 2013
Apigenin-loaded mixed micelles	Ethanol thin-film hydration method	178.565	5.6165 mg/mL	4.03 times	Zhang et al., 2017
Apigenin-loaded W/O/W emulsions	Multilayer emulsion	910	–	4 times	Kim et al., 2016

In the present study, we applied the liquid LAP method to prepare apigenin nanoparticles and thereby improve the solubility and bioavailability of apigenin. The different experimental parameters on particle size were optimized through central composite design (CCD) using the design-expert software. Apigenin nanosuspension was obtained under the optimum conditions, and the mannitol was chosen as cryoprotectant. Finally, apigenin nanoparticles were obtained by vacuum freeze-dry, then the characterization of the nanoparticles, including scanning electron microscopy (SEM), X-ray diffraction (XRD), differential scanning calorimetry (DSC) and thermo gravimetric (TG) was studied. Additionally, the dissolving test, the bioavailability in rats, the antitumor activity, the toxicity experiment and the solvent residue test were also studied, and analyzed in the present work.

## 2. Materials and methods

### 2.1. Materials

Apigenin (purity 98%) was purchased from Baoji Haoxiang Bio-technology Co., Ltd.; Poloxamer, mannitol, N, N-dimethylformamide (DMF), methanol and phosphoric acid were obtained from J&K Scientific Ltd. (Beijing, China). MTT (3-(4, 5-dimethylthiazol-2-yl)-2, 5-diphenyl-2 H-tetrazolium bromide; 98% purity) was supplied by Sigma-Aldrich. Deionized water was obtained by Hitech-K flow water purification system (Hitech Instruments Co., Ltd., Shanghai, China).

### 2.2. Preparation of apigenin nanoparticles

The apigenin nanoparticles were produced by the LAP technique, followed by freeze-drying. The specific preparation process is described in the supplementary material section and the experimental processes for preparation are illustrated in Fig. S1. In addition, the response surface methodology (RSM) design was operated to confirm the optimal conditions to prepare apigenin nanoparticles in the LAP process. Based on the results of preliminary experiments, the preparation process of the apigenin nanoparticles was optimized by a five factor, three-level CCD. The poloxamer concentration (mg/mL, *X_1_*), the temperature (°C, *X_2_*), the volume ratio of antisolvent to solvent (v/v, *X_3_*), the stirring time (min, *X_4_*), the apigenin concentration (mg/mL, *X_5_*) were chosen as the independent variables (Table S1). The values of the independent variables were based on preliminary tests. Particle size (*Y_1_*) of the apigenin was used as response variables. The particle size was measured by dynamic laser light scattering technique (ZetaPALS, Brookhaven, USA). Finally, the optimal operating conditions were obtained. The apigenin nanoparticles were prepared under the optimal conditions.

### 2.3. Physicochemical characterization of apigenin nanoparicles

#### 2.3.1. Morphology

Morphology of the samples was observed by a scanning electron microscope (Quanta 200, FEI; The Netherlands). The samples were mounted on aluminum stubs with double-sided carbon tape and sputter coated with gold for 4 min before observation.

#### 2.3.2. DSC and TG

DSC analysis of the samples was employed by DSC (TA instruments, model, DSC 204). The samples were heated from 40 to 400 °C at a heating rate of 10 °C/min.

TGA analysis of the samples was performed using a thermogravimetric analyzer (TGA, Diamond TG/DTA PerkinElmer, USA). The samples (2–3 mg) were heated at a temperature range of 40–600 °C. The experiments were performed with a heating rate of 10 °C/min under nitrogen atmosphere.

#### 2.3.3. Xrd

X-ray diffractograms were recorded by an X-ray diffractometer (Philips, Xper t-Pro; The Netherlands). The voltage and current using Cu Ka radiation were 35 kV and 40 mA, respectively. The samples were placed in a sample holder and the angular range was scanned from 5° to 60° of 2θ with a step size of 0.02° with a scan rate of 4°/min.

### 2.4. Saturation solubility and dissolution study

#### 2.4.1 Saturation solubility measurement

Saturation solubility of raw apigenin, apigenin nanoparticles and physical mixture of apigenin and mannitol were detected by HPLC method. Briefly, excess amount of each sample was put into 2 ml dissolution medium in a capped vial and stirred at 100 rpm for 48 h in the water bath of 37 °C. Artificial gastric juice (pH 1.2) with 0.4% Tween-80 and artificial intestinal juice (pH 6.8) were chosen as dissolution medium. After 48 h, the suspension was then centrifuged at 10,000 rpm for 10 min by a centrifugal and the supernatant obtained was diluted suitably with methanol and then injected to HPLC system. The drug concentration in the supernatant was its saturation solubility. The HPLC detection was implemented by the high-performance liquid chromatograph of WATERS (Waters Corporation, Milford, MA, USA), which was equipped with the Diamonsil C18 reverse-phase column (250 mm × 4.6 mm, 5 μm, China). The mobile phase, composed of 70% methanol, 30% water and 0.2% phosphoric acid (v/v/v), was delivered at 1 ml/min. The drug was detected at 353 nm and the injection volume was 10 μL. The experiment was conducted in triplicate.

#### 2.4.2. Dissolution test

Drug dissolution measurements of raw apigenin, apigenin nanoparticles and physical mixture of apigenin and mannitol were performed by HPLC. The studies were carried out in two dissolution mediums namely artificial gastric juice (pH 1.2) with 0.4% Tween-80 and artificial intestinal juice (pH 6.8). The speed of the paddle was set to 100 rpm and the bath temperature was maintained at 36.5 ± 0.5 °C. 2.53 mg of raw apigenin, 15.16 mg of physical mixture of apigenin and mannitol and 15.16 mg of apigenin nanoparticles (equivalent to 2.53 mg apigenin) were dispersed in dissolution medium (2 mL) and placed into a dialysis bag, respectively. The end-sealed dialysis bags were immersed in 250 mL beakers containing 200 mL artificial gastric juice. Moreover, 3.50 mg of raw apigenin, 20.99 mg of physical mixture of apigenin and mannitol and 20.99 mg of apigenin nanoparticles (equivalent to 3.50 mg apigenin) were dispersed in dialysis bags containing 2 mL of dissolution medium and then immersed in 250 mL beakers containing 200 mL artificial intestinal juice. At 0.08, 0.17, 0.33, 0.5, 1, 1.5, 2, 3, 4, 6, 8, 12, and 24 h, an amount of 1 ml of the dissolution medium was taken out and then supplemented with the same volume of dissolution medium, the sample obtained was centrifuged at 10,000 rpm for 10 min by the centrifugation and then injected to the HPLC system for drug concentration analysis. The detection conditions were the same as above. The experiment was conducted in triplicate.

### 2.5. Bioavailability test

A total of 12 Sprague-Dawley rats (200–250 g weight) were fasted for 12 h with free access to water. All animal studies were carried out according to NIH-approved protocols and in compliance with the Guide for the Care and Use of Laboratory Animals. The rats randomly divided into two groups of six rats each. In accordance with the dosage of 50 mg/kg (according to apigenin calculation), two groups of rats were respectively given raw apigenin and apigenin nanoparticles by gavage. After oral administration, the rats were anesthetized and blood samples obtained from the retro-orbital plexus at 0.08, 0.17, 0.33, 0.5, 1, 1.5, 2, 3, 4, 6, 8, 12, and 24 h, respectively, were put in the centrifuge tube containing heparin. The samples obtained were then centrifuged at 3000 rpm for 10 min, the plasma separated, collected, and stored in the refrigerator of 4 °C, and processed immediately.

For the extraction of apigenin from rat plasma, the plasma of 0.2 mL was transferred to 2 mL centrifuge tube and 0.4 mL of methanol was added to it. The mixture was vortexed for 3 min and mixed evenly, then was ultrasonically treated for 20 min and centrifuged at 12000 rpm for 10 min. After centrifugation, the supernate was obtained and analyzed by the HPLC method.

### 2.6. Antitumor activity evaluation

Anticancer activity of apigenin nanoparticles was investigated with MTT assay which evaluated metabolic activity of cells treated with the complexes. The human HCC cell line HepG2 was donated by Beina Chuanglian Biotechnology Research Institute (Beijing, China). The cells were cultured in Dulbecco’s modified Eagle’s medium, which was supplemented with 10% fetal bovine serum, 100 U/mL penicillin, and 100 mg/mL streptomycin at 37 °C in humidified atmosphere with 5% CO_2_. Cells in the logarithmic phase were collected for experiments. Logarithmic-phase cells were digested and carefully prepared into uniform cell suspension. The cell suspension was seeded into 96-well plates and cultivated in a 5% CO_2_ incubator at 37 °C for 24 h. After cell adhesion on the culture, the samples containing raw apigenin and apigenin nanoparticles with different apigenin concentration (apigenin: 1000.0, 500.0, 250.0, 125.0, 62.5, 31.25, 15.63, and 7.82 μg/mL) were added in the plates. Each concentration was repeated six times. The samples of different apigenin concentrations were dissolved by cell-culture medium and diluted appropriately. Following 48 h incubation, 10 μL of 5 mg/mL MTT was added into each well and incubated for 4 h. The supernatant was discarded, and 150 μL of DMSO was added to the wells. The plate was placed on a horizontal oscillator to enhance the solvation of formazan crystals. Absorbance value (OD) was measured by using the enzyme mark analyzer instrument (detection wavelength of 490 nm and reference wavelength of 630 nm) and compared with the blank. Tumor cell growth inhibition efficiency (%) was calculated using (OD cost of contrastive group-OD cost of medicate group/OD cost of contrastive group) × 100. This equation was also used to calculate the half maximal inhibitory concentration (IC50).

## 3. Results and discussion

### 3.1. Characterization of apigenin nanoparticles

Based on the results of the optimization experiments (Table S2, Table S3, and Fig. S2), the best model condition can be obtained through the optimization analysis of the CCD using the Design-Expert® software. The particle size of apigenin nanosuspension was about 159.2 nm under the optimum conditions. Then the apigenin nanoparticles were obtained by the freeze-drying method and the mannitol was used as a cryoprotectant. In the end, the apigenin nanoparticles obtained were characterized by SEM, XRD, DSC, TG, dissolving capability test, bioavailability test, and antitumor activity evaluation. The analysis results were summarized as follows.

#### 3.1.1. SEM analysis

**Figure 2.**
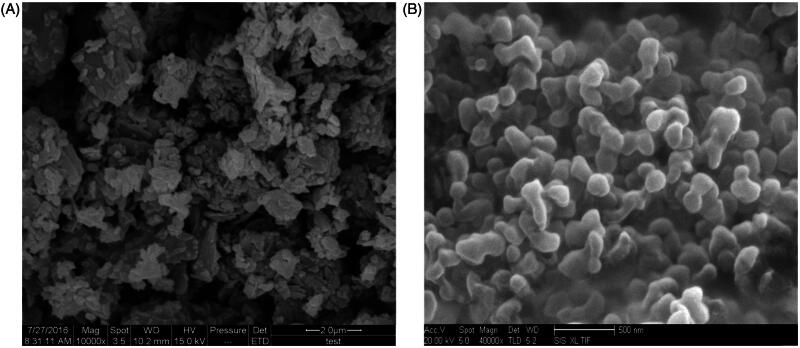
SEM images; (A) raw apigenin; (B) apigenin nanoparticles.

The results of the SEM analysis of raw apigenin and apigenin nanoparticles were shown in Figure 2. Raw apigenin exhibited irregular block with broad particle size distribution ranging between 1.06 and 6.12 μm (Figure 2A). The apigenin nanoparticles appeared as near-spherical shape, with a mean particle size of approximately 168.26 nm (Figure 2B), which was obviously different from the raw apigenin.

#### 3.1.2. Solid state analysis

**Figure 3.**
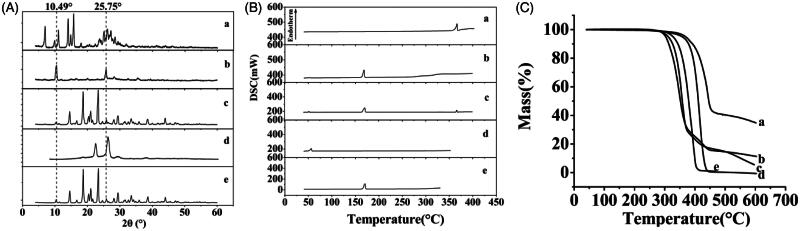
The XRD results (A) DSC results (B) and TG results (C) of each sample. (a) raw apigenin; (b) apigenin nanoparticles; (c) physical mixture of apigenin and mannitol; (d) poloxamer; and (e) mannitol.

The results of XRD and DSC of the samples, including raw apigenin, apigenin nanoparticles, physical mixture of AP and mannitol, poloxamer and mannitol were shown in Figure 3 (A and B). From the figure, Raw apigenin (Figure 3A-a) showed intense crystalline peaks, and its melting points (Figure 3B-a) were about 366.8 °C, which revealed that the drug was present as a crystalline form. Moreover, the poloxamer (Figure 3A-d) and the mannitol (Figure 3A-e) also showed intense crystalline peaks, and the melting points of the poloxamer (Figure 3B-d) and the mannitol (Figure 3B-e) were about 55.4 and 170 °C, respectively, which illustrated that the both were existed in crystal state. The physical mixture of apigenin and mannitol (Figure 3A-c) mainly displayed the characteristic peaks of mannitol, this might be because the proportion of mannitol in the physical mixture was larger, and the content of apigenin was small, resulting in the crystalline peaks of apigenin was not obvious. In addition, the DSC curve of the physical mixture (Figure 3B-c) existed three endothermic peaks at 51.8, 171.2, and 366.3 °C, the first peak could be the melting point of the poloxamer and the second and the third peaks were closer to the melting point of the mannitol and the raw apigenin, which showed that the apigenin existed as crystal in the physical mixture. While the characteristic peaks of the apigenin nanoparticles (Figure 3A-b) were less than that of the raw apigenin and the physical mixture, which indicated the crystallinity of the apigenin nanoparticles decreased significantly, while the position of the diffraction peak of the apigenin nanoparticles (2θ = 10.49° and 25.75°) was different with that of the raw apigenin, this might because the mannitol coated on the surface of the apigenin nanoparticles. In addition, the DSC curve of the apigenin nanoparticles (Figure 3B-b) only presented one endothermic melting peaks at 168.2 °C, corresponding to the melting peaks of mannitol, and the characteristic peak of the apigenin almost disappeared, consistent with the results of XRD, this illustrated that the apigenin existed as amorphism in apigenin nanoparticles. So, the apigenin nanoparticles could have better dissolution and bioavailability.

#### 3.1.3. TG analysis

The results of the TG analysis of the samples, including raw apigenin, apigenin nanoparticles, physical mixture of AP and mannitol, poloxamer and mannitol were shown in Figure 3(C). The raw apigenin (Figure 3C-a) and the physical mixture of apigenin and mannitol (Figure 3C-c) were observed to lose weight from about 354.3 °C and 308.9 °C, respectively. The poloxamer (Figure 3C-d) and the mannitol (Figure 3C-e) began to lose weight from about 343.1 °C and 315.1 °C, respectively. While the apigenin nanoparticles (Figure 3C-b) began to lose weight from about 285.6 °C. This might be due to the fact that the particle size of the apigenin nanoparticles was smaller compared with the raw apigenin led to its higher specific surface, which resulted in an easier vaporization and quickly thermal decomposition.

### 3.2. Saturation solubility and dissolution

Saturation solubility of the raw apigenin, the physical mixture of apigenin and mannitol, and the apigenin nanoparticles at 37 °C was separately 1.28 μg/mL, 13.79 μg/mL and 37.91 μg/mL in artificial gastric juice (pH 1.2), and was separately 0.81 μg/mL, 15.57 μg/mL and 52.5 μg/mL in artificial intestinal juice (pH 6.8). The equilibrium solubility of the physical mixture powder was higher than that of the raw apigenin, which illustrated that the mannitol was conducive to improving the solubility of apigenin. However, the equilibrium solubility of the apigenin nanoparticles was significantly higher than the others. In addition, based on the results of preliminary experiments, the ratio of apigenin to mannitol of 1/5 was selected as the optimal condition, and the solubility of apigenin-mannitol (1:5) nanoparticles was about 29.61 times higher than raw apigenin in artificial gastric juice (pH 1.2), and was about 64.81 times higher than raw apigenin in artificial intestinal juice (pH 6.8).

**Figure 4.**
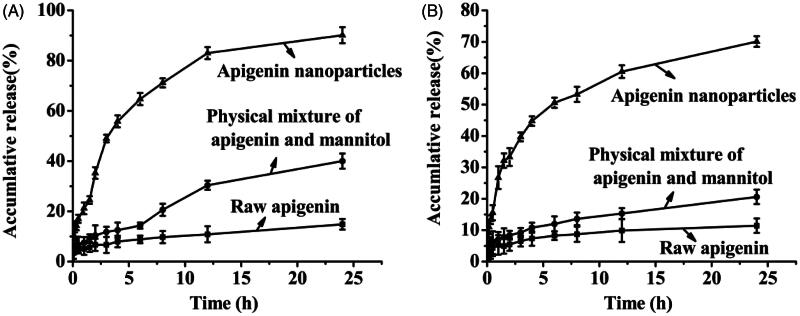
Dissolution results of the samples in artificial gastric juice (A) and artificial intestinal juice (B).

Dissolution profiles of raw apigenin, apigenin nanoparticles and physical mixture of apigenin and mannitol were illustrated in Figure 4. It was observed that the apigenin nanoparticles showed more rapid dissolution rate with much higher cumulative amount of dissolved apigenin in both dissolution mediums compared with the raw apigenin and physical mixture, and the dissolution rate of physical mixture of apigenin and mannitol was slightly higher than that of raw apigenin, this meant that the auxiliary material was conducive to improving the solubility of apigenin but it was not the key factor to enhance the solubility and the dissolution rate of apigenin. As can be seen from the figure, in artificial gastric juice (pH 1.2), 14.82% of the raw apigenin was dissolved in 24 h; while in the same period, 90.12% of the apigenin nanoparticles was dissolved. In artificial intestinal juice (pH 6.8), the apigenin nanoparticles achieving the maximum dissolution was about 70.09% at about 24 h, meanwhile, the dissolution of the raw apigenin was about 11.41%. The dissolution rate of the apigenin nanoparticles was 6.08 times that of the raw apigenin in artificial gastric juice (pH 1.2), and was 6.14 times that of the raw apigenin in artificial intestinal juice (pH 6.8). Previously, there was a related document reported that apigenin nanocrystals with the particle size of about 400–800 nm were prepared by the SAS technology (Zhang et al., 2013). In vitro dissolution study, the apigenin nanocrystals achieved a plateau of cumulative dissolution at 45 min under non-sink conditions (0.1 M HCl, 0.1 M PBS 6.8, and 0.1 M PBS 7.4), and demonstrated more than 90% of cumulative dissolution within only 20 min under sink condition (0.1 M PBS 6.8 with 0.5% Polysorbate 80). It could be seen from the above analysis that the dissolution rate of the apigenin nanoparticles in this study was slower than the apigenin nanocrystals obtained by SAS, this might be because the pretreatment of the samples were different in both studies, the apigenin nanocrystals obtained by SAS were put into the capsule and studied, however, the apigenin nanoparticles prepared in this study were placed into the dialysis bags and studied. In addition, according to Noyes–Whitney equation, with the particle size of the drugs decreasing, the specific surface area of the drugs increased accordingly, thereby increased the contact area of the solid drugs and the dissolution medium, the dissolution of the drugs was also improved accordingly (Zu et al., 2014). However, the mean particle size of the apigenin nanoparticles prepared in this study was approximately 168.26 nm, which was obviously smaller than that of the apigenin nanocrystals. Therefore, under the same treatment conditions, the apigenin nanoparticles in this study might have a faster dissolution rate compared with the apigenin nanocrystals obtained by SAS.

### 3.3. Bioavailability analysis

**Figure 5.**
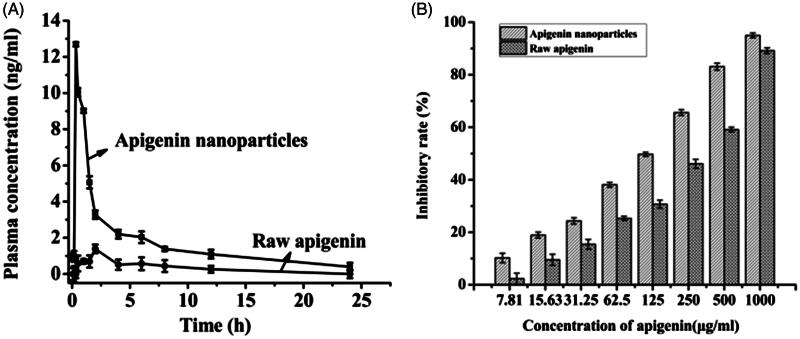
The plasma concentration (A) and the inhibitory rate (B) of raw apigenin and apigenin nanoparticles.

The bioavailability result was shown in Figure 5(A). From Figure 5(A), it was established that the plasma concentration of rats treated with apigenin nanoparticles was always higher than that of the rats treated with raw apigenin at the same dosage. The apigenin concentration in rat treated with apigenin nanoparticles and the apigenin concentration in rat treated with raw apigenin reached the maximum of 12.69 ng/mL and 1.39 ng/mL after 20 min and 120 min of taking drugs, respectively. Oral relative bioavailability of apigenin nanoparticles was calculated by comparing the corresponding AUC values of the two groups, and the results showed that the AUC values of the apigenin nanoparticles was 4.96 times higher than that of the raw apigenin. Therefore, the oral bioavailability of the apigenin nanoparticles was improved significantly compared with the raw apigenin and the significant enhancement of oral bioavailability was also consistent with the dissolution result and other characterization tests. In addition, the previous research showed that the apigenin nanocrystals obtained by SAS reached the maximum of the blood concentration after 90 min of taking drugs and its oral bioavailability increased by 3.4 times compared with that of the raw apigenin (Zhang et al., 2013), however, the apigenin nanoparticles prepared in this study reached the maximum of the blood concentration after 20 min of taking drugs and its oral bioavailability increased by 4.96 times compared with that of the raw apigenin, which illustrated that the apigenin nanoparticles can be more quickly absorbed than the apigenin nanocrystals obtained by SAS, due to its smaller particles size.

In addition, the results of toxicity experiment (Table S4) indicated that the apigenin nanoparticles and the raw apigenin had little influence on the growth of the rats, and had no toxic effect on the organs of rats.

### 3.4. Antitumor activity analysis

Apigenin was a natural flavonoid that inhibited antiproliferative activities, but was not widely used because of its low bioavailability, the experiment prepared apigenin nanoparticles by the LAP to improve the solubility of apigenin, and applied the MTT assay to compare the anticancer activity of apigenin nanoparticles and raw apigenin. In addition, it had been reported that the apigenin had an inhibitory effect on liver cancer (Li et al., 2017), so the experiment investigated the effect of drugs on the growth of HepG2 cells with different types of drugs (raw apigenin and apigenin nanoparticles). The inhibitory rates of the samples were shown in Figure 5(B). As can be seen from the figure, the inhibitory rate of all the samples increased with increasing concentration, but the apigenin nanoparticles can inhibit cell proliferation more effectively than raw apigenin. In addition, The IC50 values of apigenin nanoparticles and raw apigenin were separately 89.33 and 216.84 μg/mL, it was also seen that the apigenin nanoparticles had a higher inhibition to HepG2 cells by lower IC50 than that of raw apigenin.

In addition, the residue results of DMF in the apigenin nanoparticles (Fig. S3) indicated the residual DMF was less than the ICH limits for class II solvents of 0.088% for solvents. Therefore, the apigenin nanoparticles meet the ICH standard and are applicable for pharmaceutical use.

## 4. Conclusions

The liquid LAP technique is a quite promising, relatively simple, lower cost, and easy to operate technique which has the potential to be applied in the pharmaceutical industry. In this paper, the well water-soluble apigenin nanoparticles were prepared by the LAP method. The RSM design was used to optimize the procedure for the preparation of the apigenin nanoparticles. The apigenin nanoparticles were obtained under the optimum conditions. The physical and chemical properties of the apigenin nanoparticles were investigated, and the results showed that the apigenin nanoparticles exhibited near-spherical shape and could be converted into an amorphous form. Additionally, the data of the dissolving capability test showed that the solubility of the apigenin nanoparticles was obviously improved and were about 29.61 times and 64.81 times of the raw apigenin in artificial gastric juice and in artificial intestinal juice, respectively, and the apigenin nanoparticles showed higher dissolution rates compared to raw apigenin, and was about 6.08 times and 6.14 times than that of the raw apigenin in artificial gastric juice and in artificial intestinal juice. The oral bioavailability of apigenin nanoparticles was about 4.96 times higher than that of the raw apigenin, but the apigenin nanoparticles had no toxic effect on the organs of rats. Moreover, the apigenin nanoparticles had a higher inhibition to HepG2 cells by lower IC50 than that of raw apigenin. The residual DMF of the apigenin nanoparticles was less than the ICH limits for class II solvents of 0.088% for solvents and could be used for pharmaceutical. Therefore, this study offered a new and simple approach to improve the solubility and bioavailability of apigenin.

## Supplementary Material

IDRD_Fu_et_al_Supplemetal_Content.doc
