# Supplementary material for: Preparation, characterization and antitumor activity evaluation of apigenin nanoparticles by the liquid antisolvent precipitation technique
Source: Drug Deliv. 2017 Nov 8;24(1):1713–20. doi: 10.1080/10717544.2017.1399302 (PMC8241174; doi:10.1080/10717544.2017.1399302)
Supplement: IDRD_Fu_et_al_Supplemetal_Content.doc [file IDRD_A_1399302_SM2533.doc]

Supplementary Material

**Title:** Preparation, characterization and antitumor activity evaluation of apigenin nanoparticles by the liquid antisolvent precipitation technique.

^1^Weiwei Wu, ^2^Yuangang Zu, ^2^Li Wang, ^2^Lingling Wang, ^2^Huimei Wang, ^2^Yuanyuan Li, ^2^Mingfang Wu, ^2^Xiuhua Zhao*, ^2^Yujie Fu*.

^1^Key Laboratory of Saline-alkali Vegetation Ecology Restoration in Oil Field (SAVER), Ministry of Education, Alkali Soil Natural Environmental Science Center (ASNESC), Northeast Forestry University, Harbin Hexing Road, Harbin 150040, China

^2^Key Laboratory of Forest Plant Ecology, Northeast Forestry University, Ministry of Education, Harbin 150040, Heilongjiang, China

* Corresponding author: Xiuhua Zhao

Tel.: +86-451-82191517;

Fax: +86-451-82102082;

E-mail address: xiuhuazhao@nefu.edu.cn (Xiuhua Zhao).

* Corresponding author: Yujie, Fu

E-mail address: yujie_fu@163.com

***1. Preparation of apigenin nanoparticles***

The apigenin nanoparticles were produced by the LAP technique, followed by freeze drying. In brief, an amount of apigenin was completely dissolved in DMF at ambient temperature. The obtained solution was then injected into the deionized water containing an amount of poloxamer as antisolvent under rapid magnetic stirring at 1000 rpm by a peristaltic pump. After a period of time, the nanosuspension obtained was centrifuged at 10,000 rpm for 5 min and washed twice with a small amount of deionized water containing poloxamer to remove completely the DMF (The concentration of poloxamer was about 3 mg/ml), this was because the poloxamer could effectively prevent the growth and the aggregation of the particles. Then the freshly formed nanoparticles were redispersed in deionized water with an amount of mannitol as cryoprotectants (apigenin: mannitol, 1:5, mg/mg) and mixed well with a lower speed by the adjustable speed homogenate machine. Finally, the apigenin nanoparticles were obtained by the lyophilizer at -50 °C for 64 h. The experimental processes for preparation are illustrated in Fig. S1.


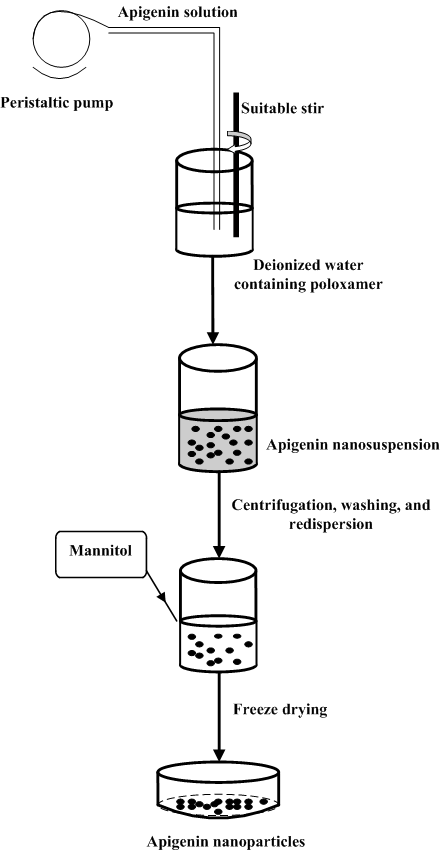


Fig. S1. Diagram of the experimental processes to prepare the apigenin nanoparticles.

***2. Optimization of the LAP process***

The Response Surface Methodology (RSM) design was operated to confirm the optimal conditions to prepare apigenin nanoparticles in the LAP process. Based on the results of preliminary experiments, the above preparation process of the apigenin nanoparticles was optimized by a five factor, three-level central composite design (CCD). The poloxamer concentration (mg/mL, *X_1_*), the temperature (°C, *X_2_*), the volume ratio of antisolvent to solvent (v/v, *X_3_*), the stirring time (min, *X_4_*), the apigenin concentration (mg/mL, *X_5_*) were chosen as the independent variables (Table S1). The values of the independent variables were based on preliminary tests. Particle size (*Y_1_*) of the apigenin was used as response variables. The particle size was measured by dynamic laser light scattering technique (ZetaPALS, Brookhaven, USA). Lastly, the optimal operating conditions were obtained. The nanosuspension obtained was centrifuged at 10,000 rpm for 5 min under the optimum condition and washed twice with deionized water containing poloxamer to remove completely the DMF. Then the nanoparticles obtained were redispersed in deionized water with an amount of mannitol as cryoprotectants (apigenin: mannitol, 1:5, mg/mg) and mixed well with a lower speed by the adjustable speed homogenate machine. Finally, the apigenin nanoparticles were obtained by the lyophilizer at -50 °C for 64 h. The apigenin nanoparticles obtained were used to carry out the following detection. In addition, the physical mixture of apigenin and mannitol in this paper was actually composed of apigenin, poloxamer and mannitol according to the corresponding proportion of the apigenin nanoparticles.

Table S1 Factors and their levels in RSM designs

| **Independent Variables** | **Coded Levels** | | | |
| --- | --- | --- | --- | --- |
|  | Symbol | -1 | 0 | 1 |
| Poloxamer concentration (mg/mL) | *X_1_* | 3.00 | 4.00 | 5.00 |
| Temperature (°C) | *X_2_* | 5.00 | 12.50 | 20.00 |
| Volume ratio of antisolvent to solvent (v/v) | *X_3_* | 2.50 | 11.25 | 20.0 |
| Stirring time (min) | *X_4_* | 3.00 | 7.50 | 12.00 |
| Apigenin concentration (mg/mL) | *X_5_* | 10.00 | 30.00 | 50.00 |

In this paper, the RSM design was used to study the effects of each variable and the interactive effects of the independent variables on the particle size. The experimental design and response values were shown in Table S2. The experimental data were regression-fitted using the Design-Expert® software, and the quadratic models were chosen as a suitable statistical model for the optimization of the particle size. The lack of fit of the model was statistically insignificant, as shown in Table S3. And the quadratic regression equation was obtained with the particle size as the objective function:

Particle size=+227.09-26.93 X_1_-56.51 X_2_+51.29X_5_+33.90 X_1_ X_2_+66.99 X_5_^2^ (1)

Table S2 Experimental design and results of the central composite design

| **Run** | **Variables** | | | | | **Responses** |
| --- | --- | --- | --- | --- | --- | --- |
|  | ***X_1_*** | ***X_2_*** | ***X_3_*** | ***X_4_*** | ***X_5_*** | ***Y_1_* (nm)** |
| 1 | 4.42 | 15.65 | 14.93 | 5.61 | 38.41 | 202.6 |
| 2 | 4.00 | 12.50 | 11.25 | 7.50 | 30.00 | 150.0 |
| 3 | 3.58 | 9.35 | 7.57 | 9.39 | 21.59 | 304.4 |
| 4 | 3.58 | 15.65 | 14.93 | 5.61 | 21.59 | 176.6 |
| 5 | 4.00 | 12.50 | 11.25 | 7.50 | 30.00 | 210.0 |
| 6 | 3.58 | 15.65 | 7.57 | 5.61 | 38.41 | 303.4 |
| 7 | 3.58 | 15.65 | 14.93 | 5.61 | 38.41 | 196.9 |
| 8 | 4.00 | 12.50 | 11.25 | 7.50 | 30.00 | 199.3 |
| 9 | 3.58 | 9.35 | 14.93 | 9.39 | 21.59 | 280.5 |
| 10 | 4.42 | 9.35 | 7.57 | 9.39 | 21.59 | 271.1 |
| 11 | 3.58 | 15.65 | 7.57 | 9.39 | 21.59 | 159.3 |
| 12 | 4.42 | 9.35 | 14.93 | 9.39 | 21.59 | 253.6 |
| 13 | 3.00 | 12.50 | 11.25 | 7.50 | 30.00 | 301.2 |
| 14 | 4.42 | 15.65 | 14.93 | 5.61 | 21.59 | 181.2 |
| 15 | 4.42 | 15.65 | 7.57 | 9.39 | 38.41 | 280.7 |
| 16 | 3.58 | 15.65 | 7.57 | 9.39 | 38.41 | 272.3 |
| 17 | 3.58 | 15.65 | 14.93 | 9.39 | 38.41 | 217.3 |
| 18 | 4.00 | 20.00 | 11.25 | 7.50 | 30.00 | 203.7 |
| 19 | 5.00 | 12.50 | 11.25 | 7.50 | 30.00 | 247.5 |
| 20 | 4.42 | 9.35 | 14.93 | 5.61 | 38.41 | 314.5 |
| 21 | 3.58 | 9.35 | 7.57 | 5.61 | 21.59 | 300.4 |
| 22 | 4.42 | 9.35 | 7.57 | 5.61 | 38.41 | 380.5 |
| 23 | 4.42 | 15.65 | 14.93 | 9.39 | 38.41 | 197.3 |
| 24 | 4.00 | 12.50 | 2.50 | 7.50 | 30.00 | 340.1 |
| 25 | 3.58 | 15.65 | 14.93 | 9.39 | 21.59 | 173.3 |
| 26 | 4.00 | 5.00 | 11.25 | 7.50 | 30.00 | 300.2 |
| 27 | 4.00 | 12.50 | 11.25 | 12.00 | 30.00 | 199.6 |
| 28 | 4.42 | 9.35 | 14.93 | 5.61 | 21.59 | 204.4 |
| 29 | 4.42 | 15.65 | 7.57 | 9.39 | 21.59 | 130.9 |
| 30 | 4.00 | 12.50 | 11.25 | 7.50 | 50.00 | 844.5 |
| 31 | 4.42 | 9.35 | 7.57 | 5.61 | 21.59 | 327.3 |
| 32 | 3.58 | 9.35 | 14.93 | 5.61 | 38.41 | 343.1 |
| 33 | 4.42 | 15.65 | 7.57 | 5.61 | 21.59 | 177.5 |
| 34 | 3.58 | 15.65 | 7.57 | 5.61 | 21.59 | 163.2 |
| 35 | 4.42 | 9.35 | 14.93 | 9.39 | 38.41 | 233.4 |
| 36 | 4.00 | 12.50 | 11.25 | 7.50 | 30.00 | 251.8 |
| 37 | 4.00 | 12.50 | 11.25 | 7.50 | 30.00 | 260.0 |
| 38 | 3.58 | 9.35 | 14.93 | 9.39 | 38.41 | 615.6 |
| 39 | 4.00 | 12.50 | 20.00 | 7.50 | 30.00 | 396.7 |
| 40 | 4.42 | 15.65 | 14.93 | 9.39 | 21.59 | 173.3 |
| 41 | 4.42 | 9.35 | 7.57 | 9.39 | 38.41 | 267.3 |
| 42 | 4.42 | 15.65 | 7.57 | 5.61 | 38.41 | 341.9 |
| 43 | 4.00 | 12.50 | 11.25 | 7.50 | 10.00 | 455.5 |
| 44 | 4.00 | 12.50 | 11.25 | 7.50 | 30.00 | 193.0 |
| 45 | 3.58 | 9.35 | 7.57 | 5.61 | 38.41 | 450.4 |
| 46 | 3.58 | 9.35 | 7.57 | 9.39 | 38.41 | 487.7 |
| 47 | 4.00 | 12.50 | 11.25 | 7.50 | 30.00 | 280.0 |
| 48 | 3.58 | 9.35 | 14.93 | 5.61 | 21.59 | 531.7 |
| 49 | 4.00 | 12.50 | 11.25 | 7.50 | 30.00 | 175.8 |
| 50 | 4.00 | 12.50 | 11.25 | 3.00 | 30.00 | 334.2 |

**Abbreviations**: Poloxamer concentration (*X_1_*), temperature (*X_2_*), volume ratio of antisolvent to solvent (*X_3_*), stirring time (*X_4_*) and apigenin concentration (*X_5_*).

Particle size (*Y_1_*).

Table S3 ANOVA for the models predicted for each response

| **Source** | ***Y_1_*** | | | | |
| --- | --- | --- | --- | --- | --- |
|  | **SS** | **DF** | **MS** | ***F*-value** | ***P*-value** |
| Model | 5.829E+005 | 5 | 1.166E+005 | 20.08 | < 0.0001 |
| *X_1_* | 31402.15 | 1 | 31402.15 | 5.41 | 0.0247 |
| *X_2_* | 1.383E+005 | 1 | 1.383E+005 | 23.83 | < 0.0001 |
| *X_5_* | 1.140E+005 | 1 | 1.140E+005 | 19.63 | < 0.0001 |
| *X_1_ X_2_* | 36778.11 | 1 | 36778.11 | 6.34 | 0.0155 |
| *X_1_^2^* | 2.624E+005 | 1 | 2.624E+005 | 45.21 | < 0.0001 |
| Residual | 2.554E+005 | 44 | 5804.59 |  |  |
| Lack of Fit | 2.413E+005 | 37 | 6521.09 | 3.23 | 0.0548 |
| Pure Error | 14121.37 | 7 | 2017.34 |  |  |
| Cor Total | 8.383E+005 | 49 |  |  |  |

**Abbreviations**: Poloxamer concentration (*X_1_*), temperature (*X_2_*) and apigenin concentration (*X_5_*).

Particle size (*Y_1_*)

Analysis of variance (ANOVA) was applied to check the sufficiency and adequacy of the model and its results were shown in Table S3. The P-values were performed to check the significance of each of the regression coefficients and indicated the interactive effects of the independent variables. The smaller the P-values, the higher the significance of the correspond coefficient. When the values of "Prob> F" were less than 0.0500, this indicated that the model terms were significant. According to the ANOVA, the single terms of the poloxamer concentration (*X_1_*), the temperature (X_2_) and the apigenin concentration (X_5_) and the quadratic term of the poloxamer concentration (*X_1_*), and the cross term of the poloxamer concentration (X_1_) and the temperature (X_2_) had pronounced effects on the particle size. The influence of other items on the particle size was not significant. Therefore, these variables were chosen to plot the response surface for the particle size while holding the volume ratio of antisolvent to solvent (X_3_), the stirring time (X_4_), and the apigenin concentration (X_5_) at central points, 11.25, 7.5 min and 30 mg/mL, respectively. As shown in Fig. S2, the particle size had a slight decrease as the poloxamer concentration (X_1_) was increased, this demonstrated the poloxamer added as a surfactant can significantly change the particle morphology and particle size, effectively coated on the surface of the drug, and inhibited the growth of particles. In addition, the particle size decreased as the temperature (X_2_) increased, this might be because the supersaturation of the system did not change much in the temperature range of 9.35-15.65°C, but with the increase of temperature, the viscosity of the system was reduced, which was beneficial to the rapid and uniform nucleation of the drugs, thereby producing small drug particles.


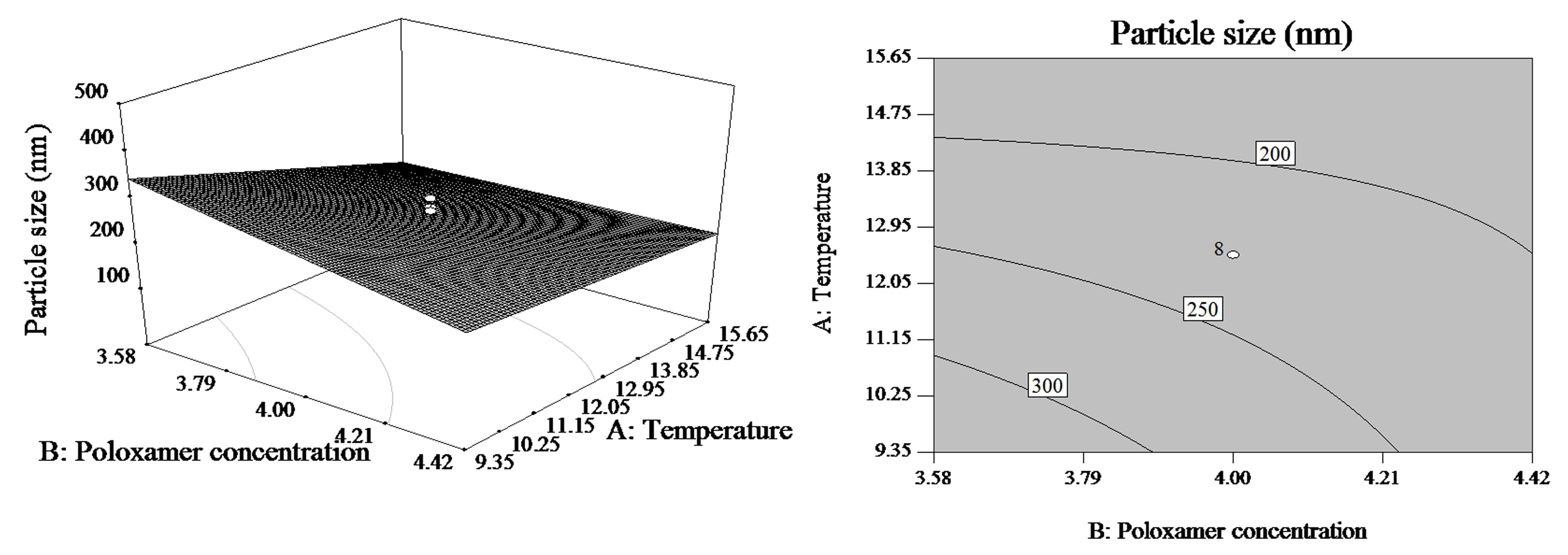


Fig.S2. Response surface plots for mean particle size of the apigenin nanoparticles showing interaction between the poloxamer concentration and the temperature. (a) 3D surface plot (b) Contour plot.

Based on the above experimental data, the best model condition can be obtained through the optimization analysis of the central composite design (CCD) using the Design-Expert® software, as follows: The poloxamer concentration was 3.58 mg/ml, the temperature was15.65°C, the volume ratio of antisolvent to solvent was 11.25, the stirring time was 7.5 min, and the apigenin concentration was 26.79 mg/ml, and the theoretical value of particle size was 153.78 nm. In order to confirm the validity of the optimization procedure, the parallel test was carried out for 3 times under the optimal conditions, and the the average particle diameter of apigenin was about 159.2 nm. Thus, the result of the response surface method was almost consistent with the actual one, and the model was reliable.

After the apigenin nanosuspension was prepared under the optimum conditions, through centrifugation separation, the apigenin nanoparticles was obtained (the average yield of nanoparticles obtained was about 79.8%±2.4% through multiple parallel experiments). Then the nanoparticles obtained were redispersed in deionized water with an amount of mannitol as cryoprotectants (apigenin: mannitol, 1:5, mg/mg) and mixed well, lastly the apigenin nanoparticles were obtained by the lyophilizer at -50 °C for 64 h. In addition, all of the following experiments were carried out using apigenin nanoparticles produced by this optimized formulation.

***3. Toxicity experiment***

Thirty-six Sprague-Dawley rats (weight, 180±20 g) were randomly divided into three groups, including control group, raw apigenin group, apigenin nanoparticles group, 12 rats in each group, male and female in half. The rats in the administration group were orally administered at a dose equivalent to 50 mg/kg of apigenin per day for 2 weeks, and the rats in control group were given the physiological saline. During this time, all rats were free to eat and drink. After 2 weeks, the rats were killed and the heart, liver, spleen, lung and kidney were taken out and weighed (accurate to 0.01). The index of immune organs was calculated according to the following formula.

Immune organ index= Weight of immune organs (g)/ Body Weigh (g) (1)

The weight gain and the immune organ index of rats in the three groups were shown in Table S4. As can be seen from the table, the body weight of the rats in all groups increased with time. The weight gain of rats in control group was about 22.48 ±4.89g, and the weight gains of rats in raw apigenin group and in apigenin nanoparticles group were 36.10 ±3.35g and 32.77 ±2.16g, respectively, which showed that the rats in the experimental group gained more weight than those in the control group. In addition, the experimental groups had no significant difference in immune organ index compared with the control group. Therefore, these results indicated that the apigenin nanoparticles and the raw apigenin had little influence on the growth of the rats, and had no toxic effect on the organs of rats.

Table S4 Effects of apigenin nanoparticles on the indices of immune organs of rats

| Group | Weight gain (g) | Cardiac organ index(mg/g) | Liver organ index(mg/g) | Spleen organ index(mg/g) | Lung organ index(mg/g) | Renal organ index(mg/g) |
| --- | --- | --- | --- | --- | --- | --- |
| Control group | 22.48 ±4.89 | 3.52 ±0.18 | 47.13 ±2.06 | 2.34 ±0.18 | 5.64 ±0.53 | 8.00 ±0.30 |
| Raw apigenin group | 36.10 ±3.35 | 3.77 ±0.13 | 49.11 ±2.52 | 2.25 ±0.18 | 5.72 ±0.60 | 8.02 ±0.40 |
| Apigenin nanoparticles group | 32.77 ±2.16 | 3.67 ±0.14 | 48.85 ±1.97 | 2.26 ±0.21 | 5.56 ±0.38 | 7.97 ±0.30 |

***4. GC measurement***

An Agilent 7890A gas chromatograph (Agilent Technologies, Palo Alto, CA, USA) was introduced to determine whether there was residual DMF in apigenin nanoparticles, equipping with a HP-5 (5% phenyl methyl siloxane) capillary column (30.0 m×320 μm×0.25 μm, nominal) and a G1540N-210 flame ionization detector. 100 mg apigenin nanoparticles were dissolved into 1 ml acetone, and was ultrasonically treated for 30 min to adequately dissolve the residual DMF and then centrifuged for 10 min at 10,000 rpm. The supernate obtained was analyzed by the GC system. GC analysis conditions of DMF were as follows: Oven temperature was initially kept at 40 °C for 5 min, and then increased at the heating rate of 30 °C/min to 240 °C, and maintained for 5 min. The injector and the detector temperatures were both set at 200 °C. The makeup gas rate was 25 mL/min, and 5 μL samples were injected manually in the GC. The rate of H_2_ and the rate of air were respectively 30 and 400 mL/min. The split ratio was 10:1.

In this study, DMF is selected as solvent, according to the ICH guidelines, DMF belongs to class 2 residual solvent, so its maximum acceptable concentration in the final product is 880 ppm. Fig. S3 showed that the results of DMF residues of the apigenin nanoparticles. Fig. S3 (a) showed the gas phase diagram of 0.088 mg/mL acetone solution (100 mg/mL acetone solution of the apigenin nanoparticles containing 0.088% DMF), and a retention time of DMF was 12.057 min. And the Fig.S3(b) showed the gas phase diagram of 100 mg/mL acetone solution of the apigenin nanoparticles. From the Fig. S3, the peak of DMF in Fig.S3(b) was significantly smaller than that of DMF in Fig. S3(a). In addition, a regression equation, Y = 493.29 x+ 6.7618 (R^2^ = 0.9995), was obtained by GC detection, and the peak area was Y and the DMF concentration was x. The linear range of DMF was 0.0125-1 mg/ml. According to the regression equation, the residual DMF content in apigenin nanoparticles was about 305 ppm. Therefore, these results revealed that the solvent residue in the apigenin nanoparticles was conformed to ICH requirements and could be used for pharmaceutical.


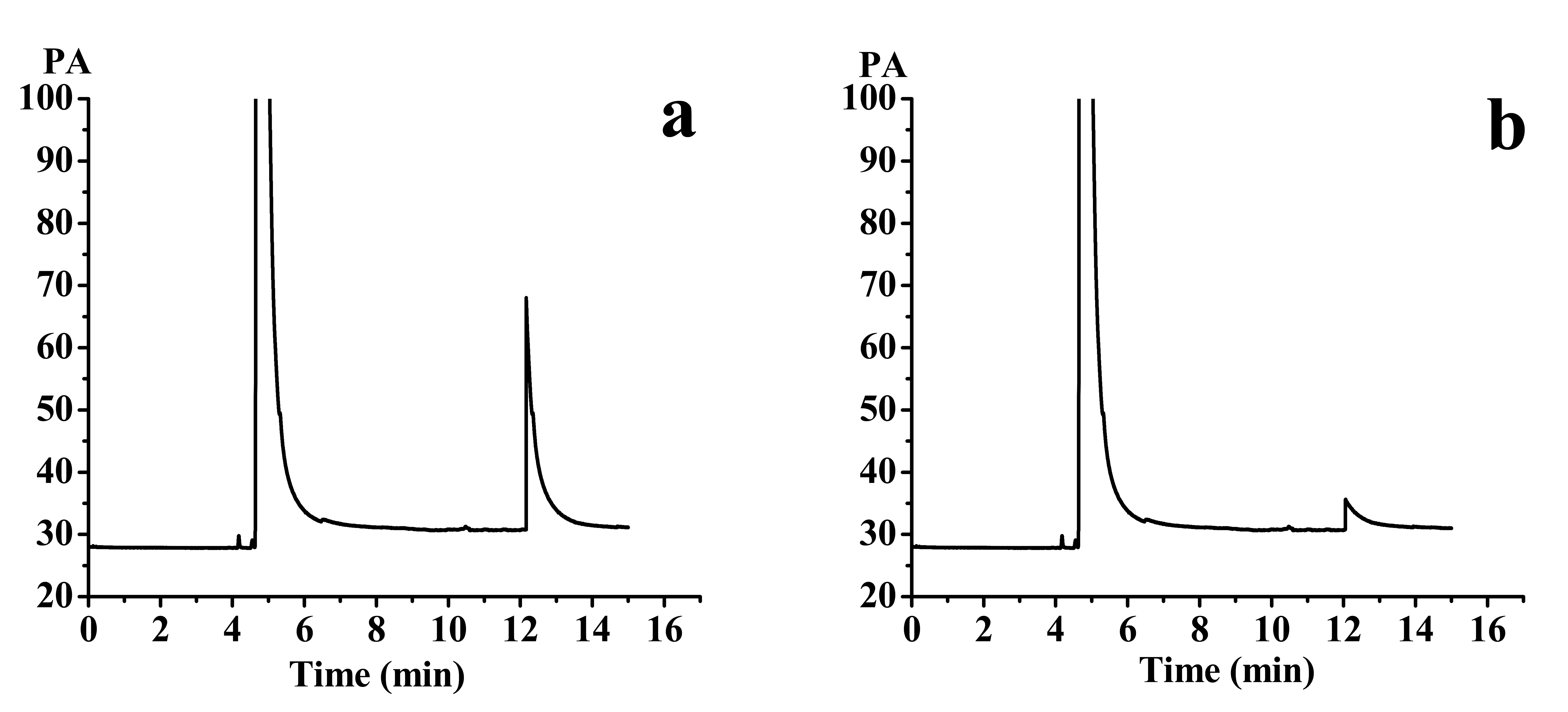


Fig.S3. (a) Gas phase diagram of 0.088 mg/mL DMF of acetone solution; (b) Gas phase diagram of 100 mg/mL acetone solution of the apigenin nanoparticles.

**References**

Dalvi SV, Dave RN. (2009). Controlling Particle Size of a Poorly Water-Soluble Drug Using Ultrasound and Stabilizers in Antisolvent Precipitation. Industrial & Engineering Chemistry Research 48; 7581-7593.
